# Radiological Innovations for Monitoring Bone Regeneration and Fracture Healing

**DOI:** 10.7759/cureus.97659

**Published:** 2025-11-24

**Authors:** Karthik D, Valeria Vanessa Varela Betancourt, Shreya Madhukar, Mainul Huda, Maria Gabriela Cerdas, Anand Sunil, Bashir Imam, Waleed Nasir, Sikandar Ali, Maahi Qureshi, Nimra Zahid, Manju Rai

**Affiliations:** 1 Sports Medicine and Arthroplasty, Manipal Hospital, Bengaluru, IND; 2 Internal Medicine, Universidad Nacional de Colombia, Bogotá, COL; 3 Medicine, Northern Lincolnshire and Goole NHS Foundation Trust, Grimsby, GBR; 4 Acute Medicine, Midland Metropolitan University Hospital, Smethwick, GBR; 5 Medicine, Universidad de Ciencias Médicas, San José, CRI; 6 Internal Medicine, Shridevi Institute of Medical Sciences and Research Hospital, Tumkuru, IND; 7 Pediatrics, University of Pittsburgh Medical Center, Coudersport, USA; 8 Internal Medicine, Sherwood Forest Hospitals NHS Foundation Trust, Sutton-in-Ashfield, GBR; 9 Internal Medicine, Doncaster and Bassetlaw Teaching Hospitals NHS Foundation Trust, Doncaster, GBR; 10 Internal Medicine, Allama Iqbal Medical College, Lahore, PAK; 11 Biotechnology, Shri Venkateshwara University, Gajraula, IND

**Keywords:** 3d printing, advanced imaging, bone regeneration, fracture healing, high-resolution mri, shear wave elastography

## Abstract

Accurate monitoring of bone regeneration and fracture healing is essential for optimizing orthopedic outcomes and guiding timely clinical interventions. While radiography, computed tomography (CT), and magnetic resonance imaging (MRI) remain standard tools, their limitations, particularly in detecting early biological activity and evaluating healing around implants, have prompted exploration of advanced imaging techniques. This narrative review was conducted through a structured search of PubMed, Scopus, and Web of Science databases for studies published between 2000 and 2025 using predefined keywords related to bone healing and imaging. Peer-reviewed original studies, systematic reviews, and clinically relevant preclinical research focusing on radiological assessment of fracture healing were included, while non-English articles, conference abstracts, and non-peer-reviewed sources were excluded. Study quality and potential sources of bias were appraised based on clarity of methodology, imaging reproducibility, and applicability to clinical practice. Recent innovations, such as high-resolution and functional MRI, quantitative CT (qCT), hybrid positron emission tomography (PET) imaging, and advanced ultrasound modalities, provide deeper insights into trabecular microarchitecture, perfusion, and osteoblastic activity. Metabolic PET tracers, radiomics, artificial intelligence (AI), and 3D printing further enhance diagnostic precision and support personalized treatment planning. Despite their promise, these modalities face challenges including high cost, limited accessibility, lack of standardization, and the need for robust clinical validation. This review summarizes the current radiological landscape, highlights emerging opportunities, and outlines future steps required for integrating advanced imaging tools into routine orthopedic practice.

## Introduction and background

Fracture healing and bone regeneration are fundamental processes in restoring skeletal function following injury. While uncomplicated fractures generally heal with minimal intervention, complex injuries such as those involving large defects, infection, or compromised vascular supply often require surgical management and are at risk for complications like delayed union or non-union, affecting up to 10% of cases [1].

Globally, fractures are a major public health concern, with an estimated 178 million new cases reported annually [2]. The incidence continues to rise due to aging populations and increasing prevalence of osteoporosis [2]. In younger adults, fractures are often trauma-related, whereas in older individuals, fragility fractures due to low bone mineral density are more common. The burden on healthcare systems is significant, particularly in cases involving the pelvis, hip, and long bones, where healing may be prolonged or incomplete [1,3].

Timely and accurate assessment of fracture healing is crucial to guide clinical decisions, avoid unnecessary surgical interventions, and optimize rehabilitation. Radiological imaging plays a central role in this evaluation. Traditional modalities such as radiography, computed tomography (CT), and magnetic resonance imaging (MRI) continue to serve as first-line tools for assessing fracture alignment, callus formation, and complications. However, each has limitations, particularly in the early detection of biological healing, evaluation around metal implants, and differentiation between viable and non-viable bone.

In response to these limitations, a growing array of advanced imaging technologies has emerged. These include high-resolution MRI, diffusion-weighted imaging (DWI), dynamic contrast-enhanced MRI (DCE-MRI), quantitative CT (qCT), micro-CT (μCT), functional ultrasound techniques such as shear wave elastography (SWE) and contrast-enhanced ultrasonography (CEUS), and hybrid imaging with positron emission tomography (PET). Innovations such as artificial intelligence (AI)-enhanced image interpretation, radiomics, and 3D printing are further augmenting diagnostic precision and enabling personalized treatment planning.

This review explores the evolving landscape of imaging modalities for monitoring bone regeneration and fracture healing. We examine the strengths, limitations, and clinical applications of both conventional and emerging techniques, highlight recent research findings, and discuss future directions for integrating these innovations into routine orthopedic practice.

## Review

Methodology

**Figure 1:**
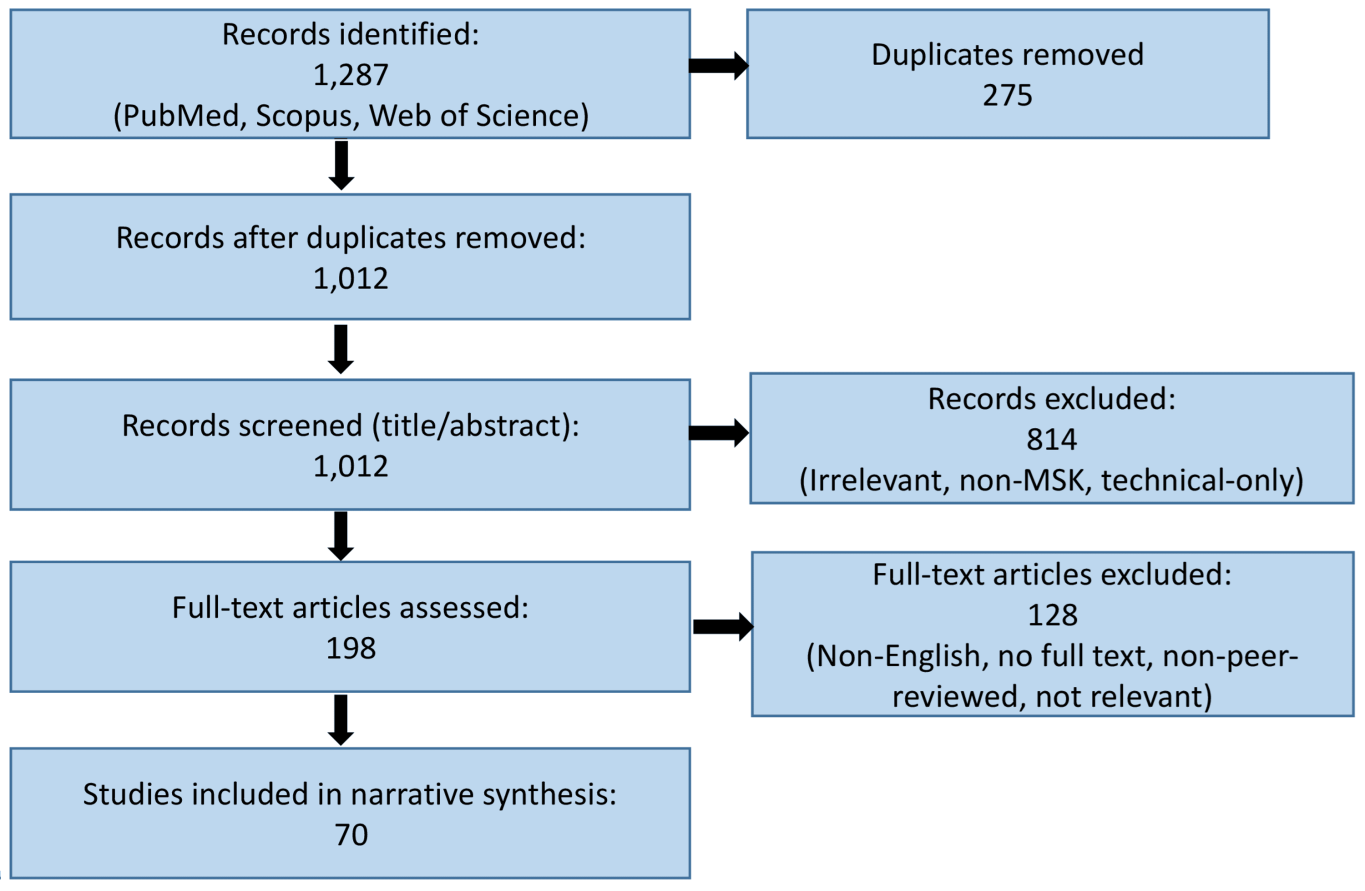
Flowchart illustrating the study selection process for the narrative review MSK, musculoskeletal

This narrative review was conducted through a comprehensive literature search of PubMed, Scopus, and Web of Science databases for studies published between 2000 and 2025 using keywords such as bone regeneration, fracture healing, advanced imaging, MRI, CT, PET, ultrasound, radiomics, and AI. Peer-reviewed original research articles, systematic reviews, meta-analyses, and relevant clinical reports focusing on radiological assessment of bone healing were included. Reference lists of key publications were manually screened to identify additional relevant studies. Priority was given to studies demonstrating methodological rigor, clinical applicability, and technological innovation. Non-English articles, conference abstracts without full text, and non-peer-reviewed sources were excluded. The selected literature was thematically analyzed to compare conventional imaging modalities with recent technological advances, emphasizing their diagnostic performance, translational potential, and clinical utility in monitoring bone regeneration and fracture healing. The study selection process is summarized in Figure 1.

Biology of bone regeneration and fracture healing - imaging-relevant aspects

Stages of Bone Healing

**Figure 2:**
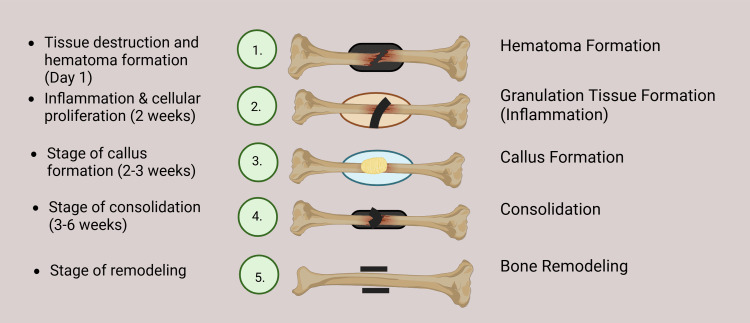
Stages of fracture healing Figure credits: Author Anand Sunil

Bone fracture healing is a highly regulated, multifaceted biological process involving both bone tissue and surrounding soft structures (Figure 2). Typically, healing proceeds through four overlapping stages: hematoma formation, granulation tissue development, callus formation, and bone remodeling [1].

The initial phase begins immediately after the fracture, marked by hematoma formation and localized inflammation. The hematoma serves as a structural and biochemical bridge that initiates healing by attracting inflammatory and repair cells [4]. Within one to two weeks, a soft callus composed of fibrous tissue and cartilage replaces the hematoma. This soft callus gradually mineralizes into a hard callus that provides temporary mechanical stability. In the final remodeling phase, osteoblasts and osteoclasts work in concert to restore the bone’s original shape and mechanical strength, a process that can span several months to years [5].

Imaging modalities are crucial for tracking these stages. In early healing, MRI and ultrasound are effective in assessing inflammation and infection. X-rays, while widely used, mainly detect soft tissue swelling or overt fractures [5]. During the callus formation stage, PET and single photon emission computed tomography (SPECT) can identify increased metabolic activity linked to osteoblast function and vascularization. Periosteal reactions and bridging of the fracture gap, key indicators of healing, are best monitored via serial X-rays. CT imaging provides higher sensitivity for early callus detection and better structural detail than standard radiographs [4].

Despite the broad availability of conventional imaging tools, their limitations in detecting early biological activity, differentiating viable from nonviable bone, and accurately evaluating healing around implants leave a critical diagnostic gap. This review aims to bridge that gap by synthesizing current evidence on both conventional and emerging radiological modalities for monitoring bone regeneration and fracture healing. Specifically, it explores how recent advancements, including high-resolution MRI, qCT, functional ultrasound, and hybrid PET imaging, can overcome the shortcomings of traditional methods and provide more precise, biologically relevant insights into bone repair. By delineating these innovations, the review seeks to guide clinicians and researchers toward evidence-based integration of advanced imaging techniques into orthopedic practice.

Imaging-Detectable Biological Markers

Bone regeneration is a lifelong physiological process involving constant remodeling, where osteoblasts synthesize new bone matrix and osteoclasts resorb damaged or aged bone tissue [6]. Osteoblasts produce key molecules like collagen and osteocalcin, critical for bone matrix formation and calcium regulation, while osteoclasts release acids and enzymes to degrade bone, releasing calcium. During resorption, telopeptides such as N- and C-terminal fragments are released. Markers like osteocalcin and the C-terminal telopeptide serve as clinical indicators of bone turnover and help diagnose conditions such as osteoporosis [6].

Advanced imaging can detect surrogate markers of osteoblastic activity, including serum levels of osteocalcin, ALP, bone-specific ALP (B-ALP), procollagen type I peptides (P1NP and P1CP), osteoprotegerin (OPG), and RANKL [7]. Areas with increased osteoblastic activity can be visualized via bone scintigraphy. Hydroxyapatite deposition, a sign of mineralization, may appear as dense regions on CT or μCT scans and is commonly seen in conditions like calcific tendinitis, often resulting from local trauma or ischemia [6].

Bone turnover can be assessed using circulating biochemical markers such as osteocalcin, ALP, B-ALP, P1NP/P1CP, OPG, and RANKL, measured by enzyme-linked immunosorbent assay (ELISA) or related assays, while imaging modalities (dual-energy X-ray absorptiometry (DXA), CT, PET) provide structural and metabolic surrogates of bone remodeling (Table 1) [7]. Areas with increased osteoblastic activity can be visualized via bone scintigraphy, particularly during the reparative and remodeling stages, where osteoblast proliferation and matrix mineralization are most active. In the early inflammatory phase, MRI and PET imaging can detect hyperemia and cellular metabolism associated with osteogenic precursor recruitment. During the soft callus phase, diffusion-weighted MRI and ultrasound can visualize tissue edema and vascular ingrowth. In the hard callus and remodeling stages, hydroxyapatite deposition, a hallmark of mineralization, appears as dense regions on CT or μCT, reflecting maturation of woven to lamellar bone. Such stage-specific imaging biomarkers enhance clinical interpretation by linking metabolic and structural findings to distinct phases of bone healing, thereby improving the precision of longitudinal monitoring [6].

**Table 1:** Biomarkers and imaging across bone healing phases DXA, dual-energy X-ray absorptiometry; ELISA, enzyme-linked immunosorbent assay; CT, computed tomography; MRI, magnetic resonance imaging

Marker	Bone Regenerative Phase	Bone Remodeling Phase	References
Imaging marker	X-ray and CT scan show callus formation. MRI shows edema and evidence of bone infection	Increase in bridging cortex on X-ray and bone mineral density on DXA. ELISA for monitoring dickkopf-1 and sclerostin	[5,6]
Biomarker	ALP, bone-specific ALP (B-ALP), osteocalcin, P1CP, P1NP	CTX, dickkopf-1, sclerostin, TRAP-5b	[6,7]

Limitations of Conventional Assessment Methods

While conventional biochemical assays such as ELISA and radioimmunoassay (RIA) are widely used for detecting bone biomarkers with high sensitivity and specificity, they are resource-intensive, require specialized reagents, and may suffer from cross-reactivity [6]. High-performance liquid chromatography (HPLC) offers superior specificity but demands technical expertise and complex equipment.

Emerging biomarker-based sensors provide real-time, cost-effective monitoring but face challenges such as a lack of standardization, sensor stability, and biocompatibility. Furthermore, these advanced diagnostic tools are typically available only in specialized centers, limiting access for patients in rural or underserved areas, especially those at high risk for fractures [6].

Conventional imaging techniques - current role and limitations

Radiography

Radiography (X-ray) remains the first-line imaging modality for evaluating fracture healing due to its widespread availability, low cost, and ability to assess bone alignment and callus formation [8]. It is routinely employed in postoperative monitoring and follow-up to detect complications such as malalignment, displacement, or non-union. However, radiographs are limited in their ability to detect soft tissue changes, early-stage healing, or biomechanical bone strength. Moreover, interobserver variability in interpretation can affect diagnostic consistency, particularly in early healing phases when callus is nonmineralized and radiographically inapparent. Prospective work in tibial and forearm fractures showed radiographs missed unmineralised callus in > 40% of clinically healing cases [9]. A cohort analysing scaphoid open reduction and internal fixation (ORIF) demonstrated only moderate inter- and intraobserver agreement (κ ≈ 0.50-0.66), underlining diagnostic variability [10]. Thus, clinical findings must accompany serial radiographs to avoid false impressions of delayed union.

CT

CT offers higher spatial resolution and superior detection of cortical bridging and early callus formation, especially in complex or peri-implant fractures [11]. However, its use is limited by radiation exposure and potential metal artifacts, which can affect image interpretation [11,12]. Despite improved diagnostic confidence compared to radiographs, studies show only moderate interobserver agreement in assessing partial union [13-17]. μCT, while valuable for research into callus microarchitecture, remains restricted to preclinical settings due to its high radiation dose and limited predictive utility in vivo [18]. Nonetheless, when standard radiographs are inconclusive or complex anatomical assessment is needed, CT remains a critical adjunct tool [19].

MRI

MRI enables noninvasive assessment of early biological processes in bone healing, such as edema, perfusion, and soft callus formation, before mineralization becomes radiographically evident [20,21]. Advanced sequences, including DWI and DCE-MRI, provide quantitative insights into cellularity, vascularity, and marrow integrity - factors predictive of successful fracture union [11].

Ultrasound

Ultrasound is a noninvasive modality that uses high-frequency sound waves (typically 1-20 MHz, and up to 75 MHz in specialized applications) to visualize internal structures, offering real-time imaging without ionizing radiation [22]. Its portability, low cost, and safety profile have made it increasingly valuable in musculoskeletal and orthopedic practice, where it is used to assess early callus formation, periosteal reactions, and superficial fractures. The absorption of ultrasound waves by tissues of varying density can generate micromechanical stresses that may stimulate biochemical pathways involved in bone repair [23].

Despite these advantages, ultrasound is highly operator-dependent, performs suboptimally in deep or obese tissues due to acoustic attenuation, and cannot penetrate cortical bone to assess internal callus morphology [24]. Evidence from randomized controlled trials of low-intensity pulsed ultrasound (LIPUS) remains mixed, with some studies showing accelerated radiographic healing while others demonstrate no meaningful clinical benefit [25].

Recent radiological innovations for monitoring bone healing

Advanced MRI Techniques

MRI has progressed from a conventional diagnostic tool to a sophisticated modality capable of assessing both structural and functional aspects of bone regeneration. Unlike CT and DXA, which primarily depict mineralized tissue and later-stage healing, advanced MRI techniques enable early visualization of cellular activity, vascularity, marrow changes, and trabecular microarchitecture, key parameters in evaluating early bone repair dynamics.

High-resolution MRI has enhanced the assessment of trabecular bone architecture. Griffin et al. (2017) demonstrated that 7-Tesla (T) MRI can quantify regional differences in trabecular bone volume across skeletal sites, offering potential for detecting microarchitectural changes relevant to fracture risk and treatment response [26]. Similarly, Chang et al. (2017) showed that both 1.5T and 3T scanners achieve spatial resolution adequate for evaluating bone microstructure, presenting MRI as a meaningful adjunct or alternative to DXA in monitoring bone quality [27].

Functional MRI techniques provide biologically relevant information beyond structural anatomy. DWI has been used to quantify changes in tissue cellularity; Lee et al. (2021) observed that increases in apparent diffusion coefficient (ADC) values correlate with therapeutic response in skeletal lesions [28], while Eveslage et al. (2023) confirmed similar applicability in osteoblastic metastases [29]. These findings support the potential of DWI to detect early fracture healing changes before radiographic union becomes apparent. DCE-MRI further contributes by quantifying perfusion, an essential determinant of healing. Nicholson et al. (2021) summarized evidence showing that DCE-MRI-derived perfusion metrics can predict fracture union as early as six weeks post-surgery, providing early prognostic insights [30].

Although MRI offers advanced insights into early biological healing, limitations persist. Metallic implants, depending on composition, may cause artifacts or safety concerns; however, titanium implants are largely considered safe and produce minimal distortion, as clarified by Kim et al. (2019) [31]. Longer scan times, higher cost, and limited availability remain additional barriers to widespread use.

When compared across imaging modalities, MRI is most effective for evaluating soft-tissue, marrow, and early callus biology, while CT maintains superiority in structural resolution. PET/CT exhibits the highest sensitivity for early metabolic activity, and ultrasound offers real-time, cost-effective functional assessment. The choice of modality thus reflects a balance between diagnostic performance, accessibility, and cost, emphasizing the need for context-specific integration of advanced imaging tools into clinical practice.

qCT and μCT

Recent advancements in radiological imaging have introduced qCT as a valuable tool for assessing bone healing and regeneration. Unlike conventional CT, qCT enables 3D measurement of volumetric bone mineral density (vBMD), thereby combining anatomical visualization with quantitative data. This dual capability allows clinicians and researchers to objectively evaluate changes in bone structure and density during the healing process. A systematic review by Willems et al. (2022), which included 13 animal studies involving rats, rabbits, and sheep, demonstrated that qCT-derived parameters such as bone mineral density, callus volume, and torsional rigidity correlate strongly with histological and mechanical markers of bone healing [32]. These findings underscore qCT’s potential as a noninvasive modality for functional assessment of bone regeneration, surpassing traditional imaging like radiographs in sensitivity and detail.

μCT, on the other hand, offers ultra-high resolution imaging suitable for evaluating bone microarchitecture at a microscopic level, particularly in small animal models. μCT enables precise measurements of parameters such as bone volume fraction, trabecular thickness, and mineral content. In a study by Wee et al. (2022) using a rat femoral midshaft fracture model, a standardized μCT protocol was employed across multiple time points to capture dynamic changes during various stages of bone healing [33]. Their results showed the capability of μCT to differentiate between early-stage low-density woven bone and mature lamellar bone, providing a comprehensive spatial and temporal overview of bone regeneration.

Despite these advantages, challenges remain in translating μCT findings to human clinical settings. Due to the differences in bone size and mechanical stress responses between animals and humans, direct extrapolation of structural metrics may not be feasible [34]. Glatt et al. (2009) proposed the use of “bridging studies” that integrate μCT findings with qCT data to improve clinical translatability and validate imaging biomarkers for human bone assessment [35].

Both modalities present practical limitations. qCT involves higher radiation exposure than conventional X-rays and requires rigorous calibration to ensure accurate measurements, which may restrict its use to specialized centers [36]. While μCT is widely used in preclinical models, in vivo longitudinal scanning is limited by radiation dose and anesthesia requirements, and many studies still rely on ex vivo endpoints [10,37].

Nevertheless, qCT and μCT represent powerful complementary modalities for studying bone healing. The integration of emerging computational approaches, including AI and automated image analysis, may further enhance the utility of these techniques in both research and clinical practice [38].

PET and Hybrid Imaging (PET/CT, PET/MRI)

PET, particularly utilizing fluorine-18 (^18^F) sodium fluoride (NaF), has emerged as a valuable imaging modality in evaluating bone healing dynamics, especially in cases of delayed union or non-union. Its application has shown significant promise in reducing the incidence of revision surgeries and enhancing clinical decision-making in orthopedic trauma and arthroplasty.

A single-centre prospective feasibility study conducted at the University Medical Centre Groningen involving 18 patients and 20 fractures assessed the effectiveness of both static and dynamic ^18^F-NaF PET/CT scans in monitoring fracture healing [39]. While static PET involves capturing images at a single time point post-injection of the radiotracer, dynamic PET acquires multiple sequential images to observe tracer kinetics over time. The study found that when tracer uptake encompassed ≥ two-thirds of the bone segment, it reliably predicted healing in all cases. Notably, 72% of the patients responded to conservative treatment, and 65% avoided the need for revision surgeries. Furthermore, whole-bone tracer uptake demonstrated a statistically significant correlation with clinical outcomes (χ² = 5.62, p = 0.018).

The utility of static ^18^F-NaF PET/CT in postoperative bone healing assessment was further illustrated in a case involving segmental femoral bone transport using an intramedullary magnetic nail. At three months post-surgery, intense radiotracer uptake at the distal distraction site (maximum standardized uptake value (SUVmax) ~ 35) and moderate uptake at the proximal docking site indicated active callus formation, supporting a cautious, nonoperative monitoring approach. Continued follow-up confirmed progressive bone healing [40].

Experimental findings from a rat femoral fracture model further reinforce the diagnostic potential of PET. In this study, non-union fractures exhibited significantly reduced ^18^F-NaF uptake as early as one week post-injury compared to control subjects, highlighting PET’s ability to detect impaired healing in the early stages [41]. Additionally, the study emphasized the superiority of ^18^F-NaF over ^18^F-fluorodeoxyglucose (FDG) in detecting osteoblastic activity and early signs of non-union. A comparative overview of various radiotracers utilized in bone healing and infection imaging is presented in Table 2 to contextualize the role of ^18^F-NaF among other agents.

**Table 2:** Comparison of various radiotracers used in fracture healing SUV, standardized uptake value; PET, positron emission tomography; FDG, fluorodeoxyglucose; NaF, sodium fluoride; FCH, fluorocholine; ^18^F, fluorine-18; ^68^Ga, gallium-68; ^111^In, indium-111

Radiotracer	Mechanism of Action	Advantages	Disadvantages	Conclusion	References
^18^F-FDG	Accumulates in areas of increased glucose metabolism (inflammation, infection)	(i) High sensitivity for infection; (ii) Useful in non-union evaluation; (iii) Quantifiable (SUV measurements)	(i) Non-specific (uptake in inflammation, tumors); (ii) Expensive	Useful in detecting infection and inflammation during impaired healing	[42,43]
^18^F-NaF	Binds to bone mineral matrix at sites of bone remodeling	(i) Superior resolution with PET; (ii) High target-to-background ratio; (iii) Fast clearance	(i) Expensive; (ii) Limited availability	Best tracer for assessing dynamic bone remodeling and perfusion	[44,45]
^68^Ga-citrate	Binds to transferrin; accumulates at infection and inflammation sites	(i) Specific for chronic infection; (ii) Useful in osteomyelitis	(i) Limited utility in general healing; (ii) Slow kinetics; (iii) Lower availability	Reserved for infection imaging rather than fracture monitoring	[46,47]
^18^F-FCH	Uptake in proliferating cells; reflects osteoblastic activity	(i) Potentially more specific than FDG; (ii) Less uptake in sterile inflammation	(i) Limited clinical data; (ii) High cost; (iii) Still experimental	Promising for bone healing assessment with better specificity	[48,49]
^111^In-labeled leukocytes	Tagged leukocytes accumulate at sites of infection	(i) High specificity for infection; (ii) Good in postoperative settings	(i) Complex preparation; (ii) Time-consuming; (iii) Not suited for general healing	Gold standard for infection imaging, not for evaluating fracture union	[50,51]

A comprehensive systematic review validated the role of quantitative PET parameters such as peak standardized uptake value (SUVpeak) and dynamic imaging in evaluating bone turnover across diverse clinical settings, including spinal fusion, pseudarthrosis, and metabolic bone disorders [52]. However, the interpretation of PET findings must consider confounding factors such as the patient’s age, sex, bone type involved, and baseline physical activity, all of which can influence standardized uptake value (SUV) measurements.

Although PET-MRI studies specifically targeting bone healing remain limited, recent progress in deep learning-assisted PET/MRI reconstruction, especially in attenuation correction and quantitative accuracy, offers a promising frontier for advanced orthopedic imaging [53].

Nonetheless, the widespread adoption of PET in routine fracture healing evaluation is constrained by certain limitations. These include exposure to ionizing radiation, high costs, limited access to PET imaging facilities, and susceptibility to image distortion from metallic implants. Furthermore, the non-specific nature of tracer uptake - which may also localize in areas of inflammation - poses a risk of diagnostic ambiguity [54,55].

Functional Ultrasound and Elastography

Recent advancements in ultrasound-based imaging have expanded its role in evaluating bone healing and regeneration. One such modality, superb microvascular imaging (SMI), enables visualization of microvascular blood flow within the callus tissue without the need for contrast agents. It produces both color-coded and grayscale images, making it suitable for detecting neovascularization during early fracture repair. Another promising technique, SWE, quantifies tissue stiffness, including that of the developing bone callus, allowing dynamic assessment of biomechanical properties during healing.

Both SMI and SWE support real-time monitoring of the healing process, facilitating timely detection of bone consolidation. A rabbit model study compared SMI against CD-31 immunohistochemistry, a known marker for angiogenesis, and SWE against DXA [56]. The study demonstrated comparable performance, suggesting that these modalities may serve as reliable, non-radiation alternatives to traditional imaging in fracture assessment.

CEUS represents another innovation, utilizing microbubble contrast agents to enhance the detection of vascular perfusion. In canine models, CEUS was able to visualize periosteal vascularization from day 10 to day 48 and endosteal vascular flow from day 13 to 36 post-osteotomy - findings that were not detected using conventional Doppler ultrasound [57]. Another study in juvenile canines reported CEUS-detected vascular signals as early as day 7 post-fracture, with peak perfusion at day 35, followed by a gradual decline corresponding to healing progression [58]. These findings underscore the sensitivity of CEUS in capturing microvascular changes, making it potentially valuable for identifying healing milestones or complications.

Beyond perfusion assessment, ultrasound radiation force (URF) techniques offer quantitative insights into the mechanical behavior of bone. By applying vibrational stimuli, URF measures resonance frequencies that vary with bone structural integrity. A pediatric study demonstrated an 80% detection rate of clavicle fractures and 70% in ulna fractures using this approach, with URF effectively distinguishing between intact and healing bone through differences in wave propagation velocity [59]. These tools could assist clinicians in identifying fracture union endpoints and optimizing treatment duration and hardware removal timing.

Photoacoustic imaging, a hybrid technique that detects ultrasound signals generated from tissue components after laser-induced heating, adds another dimension by measuring oxygenation status within the healing environment. Specifically, it differentiates between oxygenated and deoxygenated hemoglobin, offering insights into tissue viability. A murine study integrating photoacoustic and high-resolution ultrasound imaging revealed lower oxygen saturation and hemoglobin levels in non-union fractures compared to healing ones, suggesting a deteriorating microvascular environment in the former [60]. This modality may guide clinical decisions in managing delayed or impaired bone healing.

While most evidence is derived from animal studies, SWE and CEUS currently show the greatest translational potential. The selection of technique should be guided by clinical availability and operator expertise, with further validation in human studies warranted to facilitate their integration into routine orthopedic care. 

Emerging Techniques

Advances in imaging technology continue to play a transformative role in improving fracture diagnosis, treatment planning, and monitoring of bone healing. Innovative modalities such as AI-enhanced image analysis, radiomics, and 3D printing derived from imaging data offer promising improvements in diagnostic precision, workflow efficiency, and individualized treatment strategies [61].

AI, particularly deep learning algorithms, has demonstrated notable success in interpreting radiological images such as X-rays, CT scans, and MRIs. These systems enhance diagnostic accuracy and reduce reporting time. For instance, studies have shown that AI-assisted interpretation decreased the average reading time by approximately 6.3 seconds while increasing fracture detection rates by more than 10.4%, thereby minimizing the risk of overlooked injuries and improving diagnostic efficiency in clinical settings [62,63]. Additionally, research has shown that deep learning models, even when trained on small datasets or non-medical images, accurately identified fractures such as distal radius injuries, highlighting their adaptability and diagnostic potential [64]. Nonetheless, the integration of AI in routine clinical practice faces hurdles, including data privacy concerns, a lack of standardized protocols, and regulatory approval challenges [61]. Additionally, there is a risk of algorithm bias and domain shift when models are trained on single-center or homogeneous datasets.

Radiomics refers to the high-throughput extraction of quantitative features from medical images, including shape, texture, and intensity, which can be correlated with clinical outcomes. When combined with clinical and laboratory data, radiomic models have shown potential in enhancing fracture risk stratification, particularly among osteoporotic populations, thus enabling personalized care strategies [65]. However, the clinical application of radiomics remains in early phases, primarily due to technical challenges. These include variability in imaging protocols, lack of standardization, and limited multicentric validations, which currently limit its broader generalizability [66]. 

Beyond improving fracture detection accuracy, AI and radiomics are increasingly applied to predict bone-healing trajectories and assess fracture union probability. Machine learning algorithms trained on serial radiographs and CT scans have been used to estimate callus maturity and predict time to union with high accuracy. For instance, a study developed a multimodal machine learning model that integrated postoperative X-ray radiomics features with patient-reported outcome measures to predict one-year satisfaction following distal radius fracture fixation [67]. The integrated model achieved superior predictive accuracy (area under the curve (AUC) = 0.823 on the test set) compared to the radiomics-only model, demonstrating strong potential for personalized postoperative management and improved patient-centered care. Similarly, a retrospective study developed and validated a combined CT and MRI radiomics-based model to predict conservative treatment failure in postmenopausal women with osteoporotic vertebral compression fractures [68]. The integrated clinical-radiomics model achieved the highest predictive performance (AUC = 0.859), outperforming clinical (AUC = 0.684) and radiomics-only (AUC = 0.812) models, demonstrating strong potential for early identification of high-risk patients who may benefit from timely surgical intervention.

Notably, 3D printing allows the conversion of digital imaging data into tangible models that replicate patient-specific anatomy with high precision. In orthopedic surgery, such models are employed for preoperative planning and the fabrication of custom implants. Virtual simulations based on X-ray data can optimize screw placement and potentially minimize intraoperative errors and complications [69]. Pediatric orthopedic studies have demonstrated that the use of 3D-printed templates in tibial fracture surgeries significantly reduced both operative time and intraoperative radiation exposure, emphasizing their accuracy and safety [70]. Despite these advantages, the high cost of materials, regulatory constraints, and challenges related to reproducibility remain as barriers that require further investigation and technological refinement. 

Detailed comparisons of radiation exposure, artifact susceptibility, and diagnostic performance for each modality are summarized in Table 3.

**Table 3:** Radiation exposure, artifact interference, and diagnostic performance of major imaging modalities in bone healing vBMD, volumetric bone mineral density; DWI, diffusion-weighted imaging; DCE, dynamic contrast-enhanced; MRI, magnetic resonance imaging; CT, computed tomography; PET, positron emission tomography; N/A, not applicable; NaF, sodium fluoride; FDG, fluorodeoxyglucose; ^18^F, fluorine-18

Modality	Radiation Exposure	Implant Artifact Susceptibility	Diagnostic Performance/Key Notes	References
X-ray radiography	Low; typically < 0.1-0.2 mSv per exam	Minimal; metallic implants cause mild streaking but mostly interpretable	Good for alignment and late-stage callus visualization; low sensitivity for early biological healing; moderate interobserver variability	[8-10]
CT	Moderate to high; depends on region (e.g., 2-3 mSv for upper limb, higher for pelvis/spine)	High susceptibility; metal implants produce beam-hardening and streak artifacts that can reduce diagnostic accuracy	Superior for cortical bridging, early callus visualization, and peri-implant assessment; improved diagnostic confidence but only moderate agreement in partial union assessment	[11,14-18]
Quantitative CT (qCT)	Higher than conventional CT due to calibration requirements	Similar to CT; artifacts may affect density measurements	Enables vBMD assessment and quantitative callus characterization; strong correlation with mechanical and histological metrics in preclinical models	[32,36]
Micro-CT (μCT)	Very high; typically unsuitable for human in vivo imaging	N/A for clinical implants (mostly preclinical use)	Gold standard for microarchitecture assessment in animal models; excellent spatial resolution; limited clinical translation due to dose and anesthesia requirements	[13,33-35,37]
MRI	None (nonionizing)	Moderate; depends on implant material - titanium causes minimal artifact; ferromagnetic alloys may distort images	Best for early biological changes (edema, soft callus, marrow viability); advanced sequences (DWI, DCE-MRI) provide prognostic biomarkers; limited visualization of mineralized bone	[20,21,26-30,31]
PET/CT	High due to CT component and radiotracer	Artifacts from implants may distort PET attenuation correction	Highest sensitivity for metabolic activity and osteoblastic turnover; ^18^F-NaF superior to ^18^F-FDG for bone remodeling; helpful in early detection of non-union	[38-40,51-54]
Ultrasound/Elastography	None	None	Useful for early soft-callus detection, vascularity assessment, and biomechanical stiffness; operator-dependent and limited in deep tissues	[22-25,55-59]

Challenges, limitations, and future directions

Despite the promise of advanced imaging technologies in monitoring bone regeneration, several practical and methodological challenges limit their widespread clinical adoption.

Conventional modalities such as X-ray and CT remain the mainstay due to their accessibility and affordability, but they are constrained by low sensitivity in early healing, radiation exposure, and moderate interobserver variability, particularly in cases of partial union or non-union [9-17]. MRI offers superior soft tissue detail and early biological assessment, yet its utility is limited by cost, scan time, and susceptibility to artifact distortion from metal implants, although titanium-based devices are increasingly MRI-compatible [20,21,31].

Advanced tools like PET/CT and PET/MRI provide valuable insights into bone metabolism and perfusion, but face barriers including high cost, radiation burden, limited availability, and potential diagnostic ambiguity due to non-specific tracer uptake [39-41,54,55]. Similarly, ultrasound-based innovations such as CEUS, SWE, and URF show great potential in preclinical studies [56-60], but standardized protocols and robust human validation are lacking.

Emerging paradigms such as AI and radiomics offer promising automation and diagnostic enhancement, but their clinical translation is hampered by variability in imaging protocols, data privacy concerns, and insufficient multicenter validation [61-66]. The cost, reproducibility, and regulatory issues surrounding 3D printing further delay its integration into routine care [69,70].

To address these limitations, future research must prioritize multicenter longitudinal studies that validate the prognostic utility of advanced imaging modalities across diverse clinical settings. Equally important is the development of standardized acquisition and analysis protocols, particularly for technologies like AI and radiomics, where variability currently limits reproducibility and clinical translation. The integration of multimodal data - encompassing imaging findings, biochemical markers, and machine learning algorithms - will be essential to build comprehensive, predictive models that support individualized fracture management. Additionally, improving access to advanced imaging infrastructure and investing in specialized training are critical, especially in resource-constrained healthcare systems where the burden of fragility fractures continues to rise.

## Conclusions

Radiological advancements have expanded the scope of fracture healing assessment, moving beyond traditional structural imaging to include functional, metabolic, and biomechanical insights. While X-ray, CT, and MRI remain integral to clinical practice, they fall short in early detection of healing progression, especially in the presence of implants or subtle biological changes. Emerging modalities such as high-resolution MRI, DWI, PET with novel tracers, and functional ultrasound techniques offer enhanced sensitivity to tissue perfusion, osteoblastic activity, and callus biomechanics. These tools enable earlier identification of delayed union or complications and support more individualized treatment strategies.

In parallel, AI and radiomics are transforming image interpretation by extracting quantifiable biomarkers and enhancing diagnostic accuracy, while 3D printing is aiding preoperative planning through patient-specific modeling. However, challenges remain. High costs, limited access, radiation exposure, and lack of standardization continue to hinder widespread clinical implementation. Most innovations are still in preclinical or early translational phases, requiring further validation through multicenter trials. To fully realize the potential of these technologies, integration of multimodal imaging, computational analytics, and clinical data will be essential. A predictive, image-guided approach to bone healing holds promise for more precise, personalized, and outcome-driven orthopaedic care.

## References

[1] Łuczak JW, Palusińska M, Matak D (2024). The future of bone repair: emerging technologies and biomaterials in bone regeneration. Int J Mol Sci.

[2] Wu A, Bisignano C, James SL (2021). Global, regional, and national burden of bone fractures in 204 countries and territories, 1990-2019: a systematic analysis from the Global Burden of Disease Study 2019. Lancet Healthy Longev.

[3] Saul D, Khosla S (2022). Fracture healing in the setting of endocrine diseases, aging, and cellular senescence. Endocr Rev.

[4] Maruyama M, Rhee C, Utsunomiya T, Zhang N, Ueno M, Yao Z, Goodman SB (2020). Modulation of the inflammatory response and bone healing. Front Endocrinol (Lausanne).

[5] Kushchayeva Y, Pestun I, Kushchayev S, Radzikhovska N, Lewiecki EM (2022). Advancement in the treatment of osteoporosis and the effects on bone healing. J Clin Med.

[6] Naik A, Kale AA, Rajwade JM (2024). Sensing the future: a review on emerging technologies for assessing and monitoring bone health. Biomater Adv.

[7] Allende-Vigo MZ (2007). The use of biochemical markers of bone turnover in osteoporosis. P R Health Sci J.

[8] Strømmen CU, Brinch S, Hansen CF, Rasmussen JB, Ratjen U, Hansen P, Lauritzen JB (2023). Clinical and radiological signs of bone healing in conservatively treated fractures in adults. (Article in Danish). Ugeskr Laeger.

[9] Mattei L, Di Fonzo M, Marchetti S, Di Puccio F (2021). A quantitative and non-invasive vibrational method to assess bone fracture healing: a clinical case study. Int Biomech.

[10] Matzon JL, Lutsky KF, Tulipan JE, Beredjiklian PK (2021). Reliability of radiographs and computed tomography in diagnosing scaphoid union after internal fixation. J Hand Surg Am.

[11] Nicholson JA, Yapp LZ, Keating JF (2020). Monitoring of fracture healing. Update on current and future imaging modalities to predict union. Injury.

[12] Davey JS, Rotne R, Edwards G (2024). The validation of computed tomography derived radiodensity measurements of bone healing using histopathology. Injury.

[13] Hutchinson JC, Shelmerdine SC, Simcock IC, Sebire NJ, Arthurs OJ (2017). Early clinical applications for imaging at microscopic detail: microfocus computed tomography (micro-CT). Br J Radiol.

[14] Nicholson JA, Fox B, Dhir R, Simpson A, Robinson CM (2021). The accuracy of computed tomography for clavicle non-union evaluation. Shoulder Elbow.

[15] Bhattacharyya T, Bouchard KA, Phadke A, Meigs JB, Kassarjian A, Salamipour H (2006). The accuracy of computed tomography for the diagnosis of tibial nonunion. J Bone Joint Surg Am.

[16] Kleinlugtenbelt YV, Scholtes VA, Toor J (2016). Does computed tomography change our observation and management of fracture non-unions?. Arch Bone Jt Surg.

[17] Kjær M, Radev DI, Gvozdenovic R (2024). Inter- and intraobserver reliability for the computed tomography scan assessment of union after surgery for scaphoid fractures and nonunion. J Hand Surg Glob Online.

[18] Smeets R, Schöllchen M, Gauer T (2017). Artefacts in multimodal imaging of titanium, zirconium and binary titanium-zirconium alloy dental implants: an in vitro study. Dentomaxillofac Radiol.

[19] Pandey R, Raval P, Manibanakar N, Nanjayan S, McDonald C, Singh H (2023). Proximal humerus fracture(s): a review of current practice. J Clin Orthop Trauma.

[20] Schmitz N, Timmen M, Kostka K (2020). A novel MRI compatible mouse fracture model to characterize and monitor bone regeneration and tissue composition. Sci Rep.

[21] Hackenbroch C, Merz C, Palm HG, Friemert B, Stuby F, Lang P (2020). Magnetic resonance imaging in pelvic fractures - part 2: gaining information and clinical therapeutic relevance. Z Orthop Unfall.

[22] Patey SJ, Corcoran JP (2021). Ultrasound imaging. Anaesth Intensive Care Med.

[23] Claes L, Willie B (2007). The enhancement of bone regeneration by ultrasound. Prog Biophys Mol Biol.

[24] Yang T, Jin Y, Neogi A (2023). Acoustic attenuation and dispersion in fatty tissues and tissue phantoms influencing ultrasound biomedical imaging. ACS Omega.

[25] Watanabe Y, Matsushita T, Bhandari M, Zdero R, Schemitsch EH (2010). Ultrasound for fracture healing: current evidence. J Orthop Trauma.

[26] Griffin LM, Honig S, Chen C, Saha PK, Regatte R, Chang G (2017). 7T MRI of distal radius trabecular bone microarchitecture: how trabecular bone quality varies depending on distance from end-of-bone. J Magn Reson Imaging.

[27] Chang G, Boone S, Martel D (2017). MRI assessment of bone structure and microarchitecture. J Magn Reson Imaging.

[28] Lee JH, Yoo GS, Yoon YC, Park HC, Kim HS (2021). Diffusion-weighted and dynamic contrast-enhanced magnetic resonance imaging after radiation therapy for bone metastases in patients with hepatocellular carcinoma. Sci Rep.

[29] Eveslage M, Rassek P, Riegel A, Maksoud Z, Bauer J, Görlich D, Noto B (2023). Diffusion-weighted MRI for treatment response assessment in osteoblastic metastases - a repeatability study. Cancers (Basel).

[30] Nicholson JA, Yapp LZ, Keating JF, Simpson AH (2021). Monitoring of fracture healing. Update on current and future imaging modalities to predict union. Injury.

[31] Kim YH, Choi M, Kim JW (2019). Are titanium implants actually safe for magnetic resonance imaging examinations?. Arch Plast Surg.

[32] Willems A, Iҫli C, Waarsing JH, Bierma-Zeinstra SM, Meuffels DE (2022). Bone union assessment with computed tomography (CT) and statistical associations with mechanical or histological testing: a systematic review of animal studies. Calcif Tissue Int.

[33] Wee H, Khajuria DK, Kamal F, Lewis GS, Elbarbary RA (2022). Assessment of bone fracture healing using micro-computed tomography. J Vis Exp.

[34] Romanowicz GE, Zhang L, Bolger MW, Lynch M, Kohn DH (2024). Beyond bone volume: understanding tissue-level quality in healing of maxillary vs. femoral defects. Acta Biomater.

[35] Glatt V, Kwong FN, Park K (2009). Ability of recombinant human bone morphogenetic protein 2 to enhance bone healing in the presence of tobramycin: evaluation in a rat segmental defect model. J Orthop Trauma.

[36] Najjar R (2023). Radiology's ionising radiation paradox: weighing the indispensable against the detrimental in medical imaging. Cureus.

[37] Lappalainen OP, Karhula SS, Haapea M (2016). Micro-CT analysis of bone healing in rabbit calvarial critical-sized defects with solid bioactive glass, tricalcium phosphate granules or autogenous bone. J Oral Maxillofac Res.

[38] Xiao P, Zhang T, Dong XN, Han Y, Huang Y, Wang X (2020). Prediction of trabecular bone architectural features by deep learning models using simulated DXA images. Bone Rep.

[39] van Rijsewijk ND, Lanz P, Wouthuyzen-Bakker M, Glaudemans AW, IJpma FF (2025). Evaluating bone healing with [18F]NaF PET/CT during bone segment transport in femoral fracture treatment. J Nucl Med.

[40] van Rijsewijk ND, Wouthuyzen-Bakker M, van Snick JH, van Sluis J, Duis KT, Glaudemans AW, IJpma FF (2025). [18F]NaF PET/CT to assess bone healing capacity in orthopaedic trauma surgery: a feasibility study. Eur J Nucl Med Mol Imaging.

[41] Hsu WK, Feeley BT, Krenek L, Stout DB, Chatziioannou AF, Lieberman JR (2007). The use of 18F-fluoride and 18F-FDG PET scans to assess fracture healing in a rat femur model. Eur J Nucl Med Mol Imaging.

[42] Zhuang H, Alavi A (2002). 18-fluorodeoxyglucose positron emission tomographic imaging in the detection and monitoring of infection and inflammation. Semin Nucl Med.

[43] Love C, Tomas MB, Tronco GG, Palestro CJ (2005). FDG PET of infection and inflammation. Radiographics.

[44] Puri T, Frost ML, Cook GJ, Blake GM (2021). [18F] sodium fluoride PET kinetic parameters in bone imaging. Tomography.

[45] Grant FD, Fahey FH, Packard AB, Davis RT, Alavi A, Treves ST (2008). Skeletal PET with 18F-fluoride: applying new technology to an old tracer. J Nucl Med.

[46] Auletta S, Varani M, Horvat R, Galli F, Signore A, Hess S (2019). PET radiopharmaceuticals for specific bacteria imaging: a systematic review. J Clin Med.

[47] Li N, Zhu W, Zuo S, Jia M, Sun J (2003). Value of gallium-67 scanning in differentiation of malignant tumors from benign tumors or inflammatory disease in the oral and maxillofacial region. Oral Surg Oral Med Oral Pathol Oral Radiol Endod.

[48] Beheshti M, Rezaee A, Geinitz H, Loidl W, Pirich C, Langsteger W (2016). Evaluation of prostate cancer bone metastases with 18F-NaF and 18F-fluorocholine PET/CT. J Nucl Med.

[49] Chu KK, Chan AC, Ma KW (2021). Role of C11-FDG dual-tracer PET-CT scan in metastatic screening of hepatocellular carcinoma-a cost-effectiveness analysis. Hepatobiliary Surg Nutr.

[50] Heston TF, Singh C, Rout P (2025). Indium-111 white blood cell scan. StatPearls [Internet].

[51] Palestro CJ (2014). Nuclear medicine and the failed joint replacement: past, present, and future. World J Radiol.

[52] Singh SB, Gandhi OH, Shrestha BB (2025). [18F]NaF PET/CT Imaging of iliac bones to assess bone turnover. Mol Imaging Biol.

[53] Beheshti M, Mottaghy FM, Paycha F (2015). (18)F-NaF PET/CT: EANM procedure guidelines for bone imaging. Eur J Nucl Med Mol Imaging.

[54] Abdoli M, de Jong JR, Pruim J, Dierckx RA, Zaidi H (2011). Reduction of artefacts caused by hip implants in CT-based attenuation-corrected PET images using 2-D interpolation of a virtual sinogram on an irregular grid. Eur J Nucl Med Mol Imaging.

[55] Wenter V, Albert NL, Brendel M (2017). [18F]FDG PET accurately differentiates infected and non-infected non-unions after fracture fixation. Eur J Nucl Med Mol Imaging.

[56] Machado P, Blackman R, Liu JB, Dempsey C, Forsberg F, Fox T (2025). Evaluating bone fracture healing in a rabbit model using Doppler imaging modes, shear wave elastography, X-ray, and dual-energy X-ray absorptiometry. J Ultrasound Med.

[57] Jeon S, Jang J, Lee G, Park S, Lee SK, Kim H, Choi J (2020). Assessment of neovascularization during bone healing using contrast-enhanced ultrasonography in a canine tibial osteotomy model: a preliminary study. J Vet Sci.

[58] Macrì F, Angileri V, Russo T, Russo MT, Tabbì M, Di Pietro S (2021). Evaluation of bone healing using contrast-enhanced ultrasonography in non-operative treatment of tibial fracture in a puppy dog. Animals (Basel).

[59] Ghavami S, Gregory A, Webb J (2019). Ultrasound radiation force for the assessment of bone fracture healing in children: an in vivo pilot study. Sensors (Basel).

[60] Menger MM, Körbel C, Bauer D (2022). Photoacoustic imaging for the study of oxygen saturation and total hemoglobin in bone healing and non-union formation. Photoacoustics.

[61] Kuo RY, Harrison C, Curran TA (2022). Artificial intelligence in fracture detection: a systematic review and meta-analysis. Radiology.

[62] Guermazi A, Tannoury C, Kompel AJ (2022). Improving radiographic fracture recognition performance and efficiency using artificial intelligence. Radiology.

[63] Link TM, Pedoia V (2022). Using AI to improve radiographic fracture detection. Radiology.

[64] Kim DH, MacKinnon T (2018). Artificial intelligence in fracture detection: transfer learning from deep convolutional neural networks. Clin Radiol.

[65] Zhang H, Xu R, Guo X (2024). Deep learning-based automated high-accuracy location and identification of fresh vertebral compression fractures from spinal radiographs: a multicenter cohort study. Front Bioeng Biotechnol.

[66] Saravi B, Zink A, Tabukashvili E (2024). Integrating radiomics with clinical data for enhanced prediction of vertebral fracture risk. Front Bioeng Biotechnol.

[67] Yang C, Jia Z, Gao W, Xu C, Zhang L, Li J (2025). Combining radiomics of X-rays with patient functional rating scales for predicting satisfaction after radial fracture fixation: a multimodal machine learning predictive model. BMC Musculoskelet Disord.

[68] Zheng B, Yu P, Zhu Z, Liang Y, Liu H (2025). Predicting conservative treatment failure in postmenopausal women with osteoporotic vertebral compression fractures: a CT and MRI-based radiomics machine learning approach. Global Spine J.

[69] Xu J, Zhang G, He Z (2019). Anatomical reduction and precise internal fixation of intra-articular fractures of the distal radius with virtual X-ray and 3D printing. Australas Phys Eng Sci Med.

[70] Hui T, Wang J, Yu Y, Qiang L, Dong H, Lin W (2025). Clinical study of 3D printing template in the treatment of pediatric distal tibial epiphyseal fracture. BMC Musculoskelet Disord.

